# Growth from behind: Intercalation-growth of two-dimensional FeO moiré structure underneath of metal-supported graphene

**DOI:** 10.1038/srep11378

**Published:** 2015-06-15

**Authors:** Arjun Dahal, Matthias Batzill

**Affiliations:** 1 Department of Physics, University of South Florida, Tampa, FL 33620, USA

## Abstract

Growth of graphene by chemical vapor deposition on metal supports has become a promising approach for the large-scale synthesis of high quality graphene. Decoupling of the graphene from the metal has been achieved by either mechanical transfer or intercalation of elements/molecules in between the metal and graphene. Here we show that metal stabilized two-dimensional (2D)-oxide monolayers can be grown in between graphene and the metal substrate thus forming 2D-heterostructures that enable tuning of the materials properties of graphene. Specifically, we demonstrate the intercalation-growth of a 2D-FeO layer in between graphene and Pt(111), which can decouple the graphene from the metal substrate. It is known that the 2D-FeO/Pt(111) system exhibits a moiré-structure with locally strongly varying surface potential. This variation in the substrate surface potential modifies the interface charge doping to graphene locally, causing nanometer-scale variation in its work function and Fermi-level shifts relative to its Dirac point.

High-quality graphene can be grown on many late transition metal surfaces by chemical vapor deposition (CVD) even in ultrahigh vacuum (UHV)^1^,^2^,^3^,^4^,^5^. However, for many applications and fundamental studies of interface induced modifications of graphene, non-metal substrates are desirable. This may be achieved by ‘wet’-transfer processes of the graphene from the metal to other solution-stable substrates^4^,^6^. An alternative transfer-free approach for de-coupling graphene from the growth metal is by intercalation of atoms^7^,^8^ and molecules^9^,^10^,^11^ in between the graphene and the metal support. This has the advantage of a clean processing environment and thus preparation of well-defined interfaces, and as is discussed here, the possibility to grow special heterostructures. The successful intercalation of many metals^7^,^12^,^13^,^14^,^15^,^16^,^17^,^18^ and semiconductors^19^ has been demonstrated by vapor deposition and subsequent annealing to temperatures usually around 300 °C in UHV^14^,^17^,^20^. Intercalated metals are subsequently protected by the graphene layer from interaction with the environment under ambient conditions. For instance it has been shown that intercalated iron does not oxidize under atmospheric exposure at room temperature^14^. On the other hand oxidation of intercalated silicon as well as Al has been shown at elevated temperatures and oxygen pressures of ~3 × 10^−3^ Torr^21^,^22^. These latter studies resulted in the formation of SiO_2_ or Al_2_O_3_ dielectric films in between graphene and the metal and thus have been argued to be an approach for transfer-less fabrication of graphene on dielectric substrates. Here we describe the fabrication of a 2D-FeO layer in between vacuum-CVD grown graphene and a Pt(111) single crystal surface. The FeO monolayer is a truly 2D material with strongly varied properties compared to bulk FeO^23^. These special properties of 2D-FeO potentially enable a substrate-induced modification of the graphene electronic structure due to an in-plane superstructure.

Weak graphene-support interaction is a promising approach for modifying the materials properties of graphene while maintaining many of the desired properties of pristine graphene, like its high charge carrier mobility, which is not possible by e.g. chemical functionalization. The effect of a weak periodic potential induced in graphene by a substrate has been recently highlighted by the observation of Hofstadter’s butterfly for graphene supported on hex-BN^24^,^25^. In this van der Waals system the small 1.8% lattice-mismatch between graphene and hex-BN gives rise to a moiré-superstructure that causes the periodic modification of graphene. For graphene/hex-BN, as well as in many metal/graphene systems the periodic modification of graphene is a consequence of the varying adsorption sites of the carbon-atoms with respect to the substrate atoms and the consequent variation in the carbon-substrate interactions^26^. This results, for example, in a measurable corrugation of the graphene on hex-BN^27^ or on metal supports^28^,^29^,^30^. Similar to graphene supported on metals, 2D-FeO forms also a moiré-pattern on Pt(111) due to varying adsorption sites^23^,^31^,^32^,^33^,^34^. The FeO layer possesses a dipole moment normal to the surface, which is strongly modulated within the moiré-unit cell, giving rise to a modulated surface potential^23^,^35^. Here we aim at fabricating a heterostructure consisting of graphene and such a metal supported 2D-material. In this case we expect that the electronic structure of graphene may be locally altered *not* due to variation in the chemical interaction with the substrate but due to physical charge transfer as a consequence of the periodic surface potential of the FeO/Pt(111) substrate. Generally, interface charge doping of weakly physisorbed graphene is determined by the substrate work function^36^,^37^, thus the periodic surface potential of the 2D-FeO layer will induce a periodic doping variation in the graphene.

##  

### Brief description of 2D-FeO/Pt(111) system

The 2D-FeO layer on Pt(111) has been studied extensively and thus many details of this system are well-known^23^,^31^,^32^,^33^,^34^,^35^,^38^,^39^,^40^,^41^. The iron oxide monolayer structure on Pt(111) has been identified as an FeO bilayer with iron in contact with Pt and O at the surface^38^,^42^. The interlayer separation between Fe and O has been determined experimentally to be on average 0.68 Å, which is ~50% less than the bulk Fe-O inter-plane distance^42^. The in-plane lattice constant is 3.1 Å, only slightly larger than the corresponding bulk lattice constant of 3.0 Å. Similar to other 2D materials with strong interplanar bonds, the lattice mismatch between the 2D-material and the metal (a_Pt111_ = 2.77 Å) results in a moiré superstructure. In the case of FeO on Pt(111) a moiré periodicity of ~26 Å is measured^31^,^32^,^33^,^34^,^42^. The moiré structure defines the coincidence lattice between the Pt(111) surface and the FeO monolayer. Within the moiré unit cell the iron atoms occupy various adsorption sites with respect to the Pt-atoms, including iron located at atop sites, fcc-hollow sites, hcp-hollow sites and sites in between these high symmetry sites. Detailed DFT simulations have suggested the following atomic-scale picture^23^. The weakest interaction between Fe and the Pt-substrate is calculated for iron adsorption on atop sites. At this site the Fe-Pt distance is the longest while the Fe-O distance is the shortest, close to that computed for a hypothetical free-standing FeO layer. As a consequence of the different adsorption sites and bond lengths between Fe-Pt and Fe-O, the electronic structure of the FeO monolayer is locally modified. In particular the dipole-moment of the Fe-O bilayer varies strongly causing a surface potential variation of 0.4–0.6 eV across the moiré-unit cell. This surface potential variation has been observed experimentally by STM in field emission mode^35^ and verified by DFT simulations^23^. This extraordinary large variation of the surface potential within a 26 Å large unit cell makes this Pt supported 2D-FeO an exciting substrate for graphene. Here we demonstrate the synthesis of such a complex 2D-material heterostructure. We show that the 2D-FeO layer can be grown in between a CVD-grown graphene and the Pt(111) substrate by consecutive intercalation of iron and oxygen.

## Results and Discussion

### Preparation

**Figure 1:**
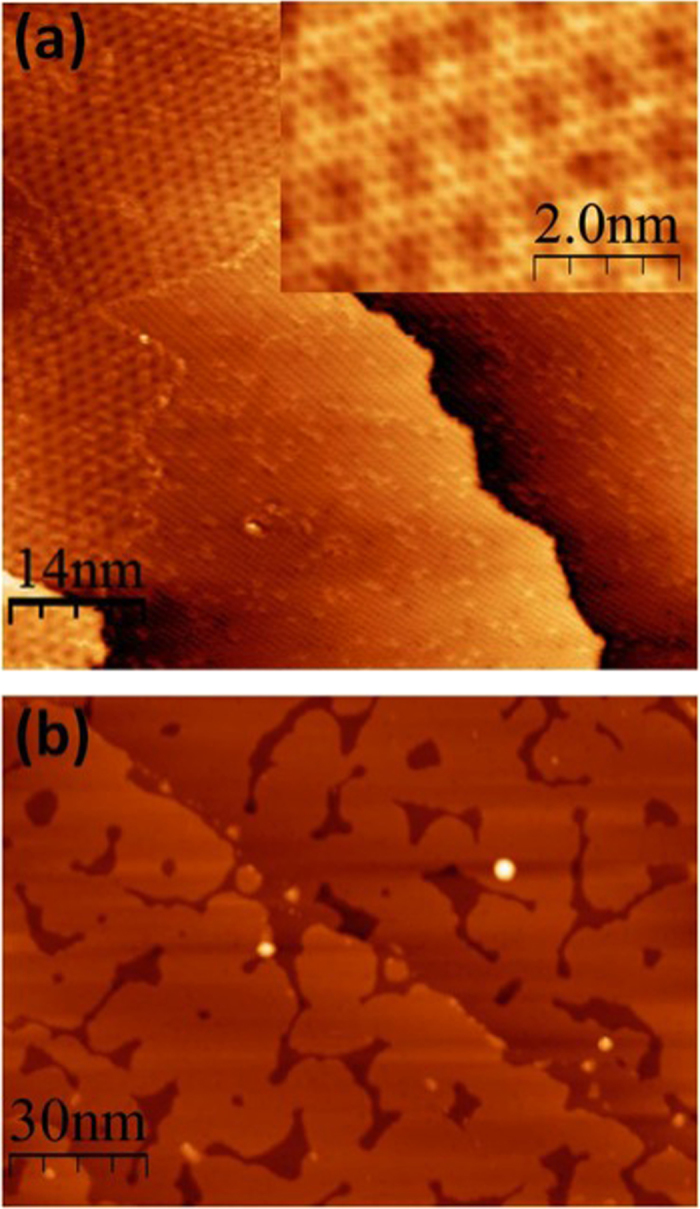
STM images of Graphene/Pt(111) and graphene/Fe/Pt(111) samples. (**a**) STM image of graphene on Pt(111) substrate. Graphene exhibits different orientional domains that give various moiré-unit cells. 3 moire domains are visible in (**a**). The inset shows a high-resolution image showing both the graphene honeycomb structure as well as the moiré superstructure. (**b**) STM image after intercalation of close to one monolayer of iron underneath graphene. As is evident from the incomplete monolayer, iron grows in an atomic-height 2D layer on Pt. STM imaging conditions: V_bias_ = 0.01 V, I_t_ = 1.0 nA (a); V _bias_ = 0.3 V, I_t_ = 1.0 nA (**b**).

Graphene grown on Pt(111) exhibits different orientational domains that give rise to various moiré-unit cells as shown on Fig. 1(a), the largest graphene moiré pattern on Pt(111) has a periodicity of 22 Å^30^. Subsequent to the preparation of graphene, iron has been deposited on the graphene/Pt(111) and the sample has been annealed to 300 °C. As previously reported for other graphene/metal systems this procedure results in an intercalation of Fe in between the graphene and the metal substrate^14^. Figure 1(b) shows a large-scale STM characterization after intercalation of close to one monolayer of iron. It is apparent that the iron grows in a layer fashion on the Pt(111) surface indicating a large iron-Pt cohesive energy. The cohesive energy between Pt and Fe is the driving force for the iron intercalation. After intercalation of iron underneath of graphene the sample can be taken out of vacuum and exposed to ambient condition without oxidation of the iron. This has been confirmed by XPS, which shows that the iron remains metallic and negligible O-1s peak. Thus consistent with previous studies graphene on metals is a good oxidation/corrosion protective coating^43^ and is consistent with the fact that graphene is impermeable to gases. However, previous studies have shown that the intercalation of molecules may proceed from edges or defects in the graphene^9^,^44^. Such a diffusion from edges requires additional activation. Our studies for exposure of graphene/Fe/Pt(111) to low (vacuum compatible) oxygen pressures of 10^−6^ Torr show that even at sample temperatures up to 250 °C the iron may not be oxidized. To achieve oxidation, the sample was transferred into a ‘high-pressure’ cell. In the high pressure cell the sample could be exposed at a much higher oxygen chemical potential by increasing the O_2_ pressure to 40 Torr and heat the sample to 235 °C. It is known that graphene remains stable at these temperatures and oxygen partial pressure, e.g. annealing of transferred graphene in air to 350 °C is frequently used for ‘burning-off’ PMMA-residue^45^. After this elevated pressure and elevated temperature processing XPS shows oxidation of the iron, as evident from the higher oxidation state of Fe (mostly 2+) (see supplement Fig. S1) and the appearance of an O-1s state.

### Scanning tunneling microscopy characterization

**Figure 2:**
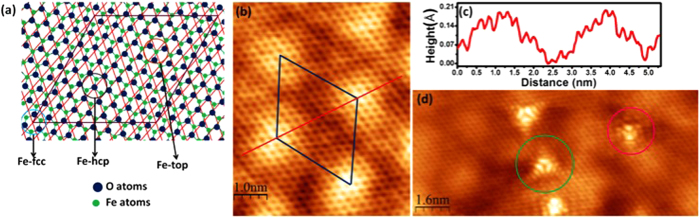
STM images of graphene/FeO/Pt(111) sample. (**a**) Top view of the previously determined FeO layer on Pt(111) forming a moiré superstructure. The red-lines indicate the Pt(111) substrate. The iron-oxygen bilayer appears hexagonal in the top view, however the iron and oxygen atoms are in different planes, with the iron closer to the Pt-substrate. (**b**) STM image of graphene on a 2D-FeO layer. The graphene honeycomb structure is resolved, while the superstructure (indicated by the unit unit cell) is due to the underlying 2D-FeO moiré-unit cell. (**c**) Cross-sectional profile along a moiré structure. (**d**) STM image of point defects in the graphene lattice observed on graphene/FeO/Pt sample. Two kinds of defects are observed: (**i**) defects with 3-fold symmetry (green circle) and defects exhibiting apparent 2-fold symmetry (red circle). These defects are due to point defects in the graphene and their structure indicates that the graphene is electronically well-decoupled from the substrate. STM imaging conditions: V_bias_ = 1.0 V, I_t_ = 1.0 nA ((**a**) and (**d**)).

To confirm that the 2D-FeO, schematically shown in Fig. 2(a), has formed at the interface between graphene and Pt(111) we performed high resolution STM studies. In addition to the graphene honeycomb structure a long range periodic surface modulation with a corrugation of about 0.2 Å can be seen in Fig. 2(b). The unit cell of this hexagonal superstructure is measured to 26 Å, which is larger than any reported graphene/Pt(111) moiré pattern^30^. The 26 Å, however, coincide with the moiré-unit cell of 2D-FeO. Furthermore, the 26 Å periodicity is the only superstructure observed on the surface, i.e. unlike the case of graphene on Pt(111) which exhibits various orientation domains with various moiré superstructures. In other words, the long-range modulation observed in STM is that of the underlying 2D-FeO/Pt(111) moiré structure and the graphene uniformly follows this undulation regardless of its relative orientation with the substrate. The absence of any substrate-induced superstructure in the graphene, indicates the lack of any chemical interactions between the graphene and the 2D-FeO and thus a decoupling of the graphene from the substrate. In addition to the perfect graphene-honeycomb we occasionally observe point defects in the graphene lattice in STM images. Point-defects were present in the graphene/Pt(111) surface prior to the FeO growth. On the Pt-surface the metal induced short-range moiré structure and electronic as well as chemical coupling of defects with the metal substrate makes the identification of the defect structure more challenging^46^. After FeO intercalation growth, the nanometer-range electronic structure variation induced in the graphene by the point defect can be clearly observed in STM. The observation of the electronic structure variations of these defects in STM is further evidence for the absence of chemical interactions between graphene and the substrate. The electronic structure variations around the defect imaged in STM are characteristic for specific point defects, which have been observed previously on epitaxial graphene, graphite, or other weakly interacting van-der Waals substrates. Also defect structures have been predicted theoretically by DFT and simulated STM images^47^,^48^. In our studies we mostly identify two defect structures shown in Fig. 2(d). One defect exhibits 3-fold symmetry (with two equivalent species rotated 60° present), while the other structure appears two-fold symmetric. This symmetry should be reflected in the structure of the point defect. The 3-fold symmetric structure has been previously identified as a single carbon vacancy^49^,^50^, while the other defect may be a bi-vacancy^51^ or a Thrower-Stone-Wales defect^52^.

### Photoemission characterization and evidence for 2D-FeO induced charge doping of graphene

In addition to STM we conducted photoemission studies. XPS shows that the iron is in a 2+ charge state. Comparison of the Fe-2p spectra for a 2D-FeO/Pt(111) film with that of the graphene covered surface are very similar, further supporting the formation of the 2D-FeO monolayer structure underneath of graphene despite the different preparation conditions that need to be employed to oxidize the iron underneath a graphene layer. A comparison of the Fe-2p spectra is shown in Fig. S1 in the supporting information.

**Figure 3:**
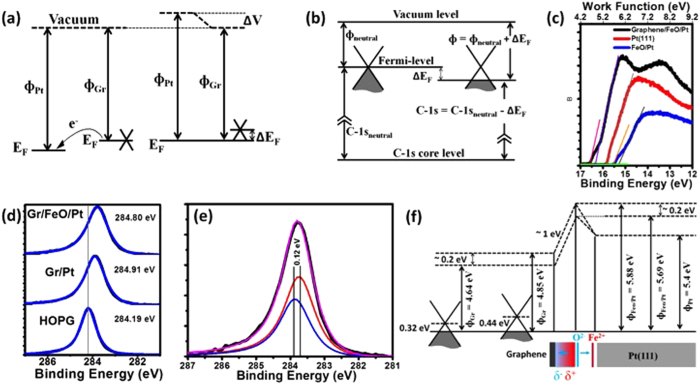
Interface band alignment measured by photoemission spectroscopy. (**a**) Schematic showing electron transfer for graphene brought into contact with a metal. Because of the low density of states at the Dirac point, this charge transfer results in a Fermi-level shift away from the Dirac point. In a rigid band model this shift of the Fermi-level also causes the work function and the C-1s core level (referenced to the Fermi-level) to shift by the same amount. This is schematically shown in (**b**). (**c**) Secondary electron cut-off in UPS measurements that enable determination of the sample work function for graphene/FeO/Pt(111), Pt(111), and FeO/Pt(111) samples. The cut-off for FeO/Pt(111) and graphene/FeO/Pt(111) cannot be explained by a single sample work function, indicating non-uniform surface potential for FeO/Pt(111) which leads to non-uniform charge transfer in graphene and thus variation in its the work function as illustrated in (**b**). The work functions of graphene/FeO/Pt(111), Pt(111), and FeO/Pt(111) samples are measured to be 4.64–4.85 eV, 5.4 eV and 5.69 eV–5.88 eV respectively. (d) XPS C-1s core-level position for HOPG, graphene/Pt(111), and graphene/FeO/Pt(111) samples. The shift in the C-1s core level away from the value of HOPG indicates a p-type doping of graphene as indicated in (**b**). For graphene on FeO/Pt(111) the peak is broadened compared to graphene/Pt. This may indicate a variation of the charge doping. (**e**) A deconvolution of the C-1s peak for graphene/FeO with two components with the same peak shape as for graphene/Pt and a peak separation deduced from the work function variation of graphene/FeO shown in (**c**). (**f**) The summary of the interface band alignment for graphene on FeO/Pt. The dipole of the 2D-FeO layer causes an increase of the surface potential of Pt. Because of the variation of FeO dipole this increase is non-uniform and we observe a variation of ~0.2 eV in this surface dipole layer. As a consequence of this variation the charge doping of graphene also varies by ~0.2 eV. The interface dipole between graphene and the substrate of ~1 eV is a consequence of charge redistribution for supported graphene versus the freestanding graphene.

To learn more about the interface charge transfer doping of graphene supported on FeO/Pt(111) we conducted work function measurements by evaluating the secondary electron-cut off in ultra violet photoemission spectroscopy (UPS) as well as detailed analysis of the C-1s peak position. Interface charge doping of graphene as a consequence of work function difference between graphene and the substrate are well documented and well understood especially for metals^36^,^53^. As illustrated in Fig. 3(a) electron transfer from graphene to the metal is required to align the Fermi-level of graphene with that of the substrate. Because of the low density of states at the Dirac point of graphene this charge transfer results in a measurable shift of the Fermi-level away from the Dirac point. This shift has been, for example, measured directly in angle resolved photoemission spectroscopy on several metals^5^,^54^. A rigid band model, i.e. a band model in which the relative energy positions of the vacuum-level, the Dirac-point, and C-1s core levels are unaffected by the Fermi-level position (charge doping of graphene) implies that a shift of the Fermi-level gives rise to equivalent shifts of the C-1s binding energy (measured relative to the Fermi-level) and the work function, which is by definition the energy difference between the vacuum level and the Fermi-level. The shift of the C-1s peak position as a function of graphene charge doping has been demonstrated before for graphene supported on different work function metals^53^. The latter, i.e. the variation of the work function of graphene as a consequence of charge doping is well-known and has been exploited in novel graphene field effect devices such as the ‘barristor’^55^ or graphene tunnel junctions transistors^56^. Figure 3(b) shows schematically the simple dependence of the work function and C-1s binding energy on the Fermi-level position in graphene, which in turn is controlled by interface charge transfer from the substrate (Fig. 3(a)). Consequently, a variation of the charge doping of graphene due to the surface potential variation in the 2D-FeO/Pt(111) substrate should be expressed in the work function as well as in the C-1s peak position of graphene. To investigate this we compared the C-1s core levels of graphene on different samples, as well as the work function of these samples by evaluating their secondary electron cut-off in UPS measurements. Figure 3(c) shows the secondary electron cut-off measured with UPS for Pt(111), FeO/Pt(111), and graphene/FeO/Pt(111). From these measurements we extract a work function of Φ_Pt_ = 5.4 eV for Pt(111), slightly less than some literature values^57^. For the FeO terminated Pt(111) surface the work function is increased to Φ_FeO/Pt_ = 5.69–5.88 eV due to the surface dipole of the FeO-layer. Importantly, rather than one sharp cut-off two cut-off energies can be discerned indicating locally varying work function of the sample. This is in agreement with the expectation that different regions within the 2D-FeO-moiré structure exhibit different surface potentials. However, we only measure a difference of ~0.2 eV between the two cut-offs, which is smaller than what has been calculated^23^ but may be a consequence of our space averaging technique. Importantly, this split in the cut-off persists for the graphene/FeO/Pt(111) sample. The work function of the graphene-terminated surface is slightly larger than that of free standing graphene, which is commonly cited to be ~4.5 eV. For the two cut-offs for the FeO-supported graphene we obtain values of Φ_graphene/FeO/Pt_ = 4.85 eV and 4.64 eV.

According to the above arguments a variations in the charge doping should also cause a variation in the C-1s core level. The C-1s peak position for HOPG, graphene on Pt(111), and graphene/FeO/Pt(111) is shown in Fig. 3(d). We use the C-1s peak for HOPG as a reference, which we assume is close to the C-1s position of charge neutral graphene. For graphene/Pt(111) micro-ARPES data have determined previously that graphene is p-doped with the Fermi-level ~0.3 eV^5^ below the Dirac point as expected for a large work function metal like Pt. This p-doping of graphene is confirmed by our C-1s XPS data that shows the peak shifted by ~0.28 eV to lower binding energy compared to HOPG. After intercalation of 2D-FeO the C-1s peak broadens slightly and this is consistent with the presence of non-uniformly doped graphene. From the above extracted work function ‘splitting’ of ~0.2 eV we expect a similar splitting of the C-1s core level (see Fig. 3(b)). A more accurate correlation based on published DFT simulations between the Fermi-level shift and the work function of the substrate is shown in the supplemental Fig. S2. This analysis shows a Fermi-level shift of 0.12 eV for a substrate work function difference of 0.2 eV. The broadened C-1s peak of graphene on FeO/Pt(111) can be well fitted with two components separated by 0.12 eV as shown in Fig. 3(e). In this fitting procedure the same peak shape and full-width-half-maximum as measured for the graphene on Pt(111) has been used for the two components. However, the small splitting of the C-1s peak into two (or several) components is at the limit of the resolution of our XPS measurement and this makes a determination of a reliable value for the variation of the charge doping in 2D-FeO supported graphene impossible. Nevertheless, both the work function measurement and the XPS data (the broadening of the C-1s peak) are consistent with a variation in the interface charge doping within the moiré-structure of graphene. Our best estimates of the interface band alignments, based on our measurements, are schematically summarized in Fig. 3(f). In the diagram in Fig. 3(f), the ~1 eV dipole, which allows the alignment of the vacuum level of substrate and graphene, is due to the so-called ‘push-back’ or ‘cushion’ effect and is a consequence of interface charge redistribution. The measured value of ~1 eV (from the secondary electron cut off work function measurement) is in reasonable agreement with calculated values of ~0.9 eV^36^ for graphene/metal interface (see Fig. S2).

### Conclusions and outlook

In a broader view, this study demonstrates the potential of interface engineering of graphene by direct growth processes without any mechanical graphene transfer. A similar 2D FeO oxide layer to that observed on Pt(111) has also been grown on other late transition metals, namely Ru(0001)^58^ and Pd(111)^59^. Especially Ru(0001) is an exciting substrate since single domain graphene is readily grown on Ru(0001) and thus successful intercalation of a 2D-FeO layer would ensure well defined orientation alignment between graphene and the 2D-FeO. Large area single crystal graphene aligned with a 2D-FeO layer is important because it would enable the use of space averaging techniques like angle resolved photoemission spectroscopy to characterize the graphene band structure and thus directly probe the influence of the support induced periodic potential on the electronic structure of graphene. The growth of 2D oxides by intercalation may not be limited to FeO. A large number of 2D-oxide monolayers on late transition metal substrates have been explored over the last decade^60^,^61^,^62^,^63^,^64^,^65^,^66^,^67^,^68^,^69^,^70^. These are the same transition metal substrates that support CVD growth of graphene^8^ and thus may allow combining oxide monolayers with graphene. Here we have shown that these oxide monolayers can be grown underneath of graphene under the right conditions. At least in the case of 2D-FeO the main change that needs to be implemented to allow the oxidation of intercalated metals beneath graphene is a higher oxygen pressure than is commonly used for fabrication of monolayer oxides in UHV. This observation paves the way of merging the graphene research with that of 2D oxide monolayers which could benefit both research fields. On the one hand, as we have demonstrated here, the graphene-properties may be tuned by the oxide monolayers. On the other hand the protective coating-properties of graphene will enable to bring the oxide monolayers out of UHV and thus make them amendable for applications and/or characterization with non-UHV methods.

## Methods

Graphene/FeO/Pt(111) interfaces were studied in two different UHV systems with base pressures of 2 × 10^−10^ Torr. The first UHV system was equipped with an Omicron VT-scanning tunneling microscope (STM) operated at room temperature. The second system was a UHV chamber for x-ray photoemission spectroscopy (XPS) and ultraviolet photoemission spectroscopy (UPS). An Mg/Al dual anode x-ray source and He-VUV lamp were used for XPS and UPS, respectively. An Omicron-Sphera II hemispherical energy analyzer was used for measuring the photoelectron spectra. Both chambers were also equipped with sample preparation equipment, including sample heating, argon-ion sputter gun, precision-leak valves for oxygen and ethylene dosing, and a mini electron-beam evaporation source (tectra GmbH) for iron deposition.

The Pt(111) sample was cleaned by repeated ion-sputtering annealing cycles. Graphene was grown on the clean Pt(111) sample by exposure to 1 × 10^−7^ Torr ethylene at 750 °C for 10 min. This procedure resulted in a full monolayer coverage of the Pt(111) sample with graphene. Several graphene domains with different rotational angles were observed, as reported previously. Iron was evaporated by electron-beam bombardment from a 4 mm-diameter iron rod inside a water-cooled copper shroud. The iron was deposited with the graphene/Pt(111) sample at room temperature. Subsequently, the Fe/graphene/Pt(111) sample was annealed in vacuum at 300 °C to intercalate Fe. The inert nature of graphene enables us to transport the graphene covered surface through air. The graphene/Fe/Pt(111) sample was transferred to an UHV-XPS system equipped with an attached high-pressure cell. The high-pressure cell contained a sample holder fitted with a ceramic button heater allowing the samples to heat in reactive gas environments. Static volumes of O_2_ were introduced in the high-pressure cell. The O_2_ pressure was monitored with an Inficon capacitance gauge. The graphene/Fe/Pt(111) sample was at room temperature when first exposed to O_2_ at 40 Torr pressure, ramped to 235 °C, and allowed to cool to below 50 °C in the oxygen atmosphere. Compositional analysis of the sample prior and after high-pressure oxygen treatment was performed with XPS without air exposure to ensure oxidation of the iron and maintenance of the graphene C-1s peak intensity. Then the sample was transferred through air for characterization by UHV-STM and detailed measurements by XPS/UPS. Prior to these measurements, the sample was annealed in UHV at 500 °C. Evaluation of the C-1s core level was done by fitting it with a Doniach–Sunjic peak shape to accommodate for the typical asymmetric line shape of sp^2^ carbon.

For comparison of the XPS/UPS spectra with a non-graphene covered surface, FeO films were prepared in-situ following standard preparation conditions for FeO/Pt(111)^34^. Briefly, Fe was deposition at room temperature on clean Pt(111) and subsequently oxidized for 2 min at 600 °C in O_2_ atmosphere at 10^−6^ Torr pressure.

### Supporting Information

(i)Fe-2p XPS data for Fe on Pt(111), FeO/Pt(111), and graphene/FeO/Pt(111): (ii) calculated relationship between work function difference between substrate and graphene and the corresponding Fermi-level shift in graphene, adapted from ref. [36].

## Additional Information

**How to cite this article**: Dahal, A. and Batzill, M. Growth from behind: Intercalation-growth of two-dimensional FeO moiré structure underneath of metal-supported graphene. *Sci. Rep*. **5**, 11378; doi: 10.1038/srep11378 (2015).

## Supplementary Material

Supplementary Information
