# Supplementary material for: Growth from behind: Intercalation-growth of two-dimensional FeO moiré structure underneath of metal-supported graphene
Source: Sci Rep. 2015 Jun 15;5:11378. doi: 10.1038/srep11378 (PMC4466883; doi:10.1038/srep11378)
Supplement: Supplementary Information [file srep11378-s1.pdf]

Supporting Information

Growth from behind: Intercalation-growth of two-dimensional FeO moiré structure underneath of metal-supported graphene

Arjun Dahal, Matthias Batzill

Department of Physics, University of South Florida, Tampa, FL 33620, USA

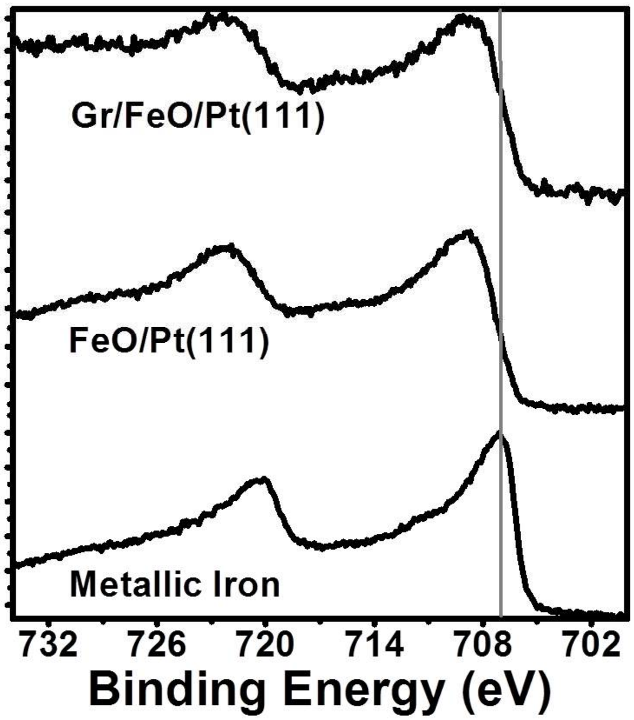

Figure S1: Comparison of Fe-2p XPS intensity for iron as deposited on Pt(111) (metallic iron), after oxidation and formation of a 2D-FeO layer on Pt(111) (FeO/Pt(111)), and for a 2D-FeO film formed underneath of graphene (Gr/FeO/Pt(111)). The Fe-2p show a very similar peak shape after oxidation, with and without graphene, indicating that in both cases the same 2D-FeO film has formed. The XPS data also in agreement with previously reported data for the 2D-FeO film, see e.g. ref. 34 in main manuscript.

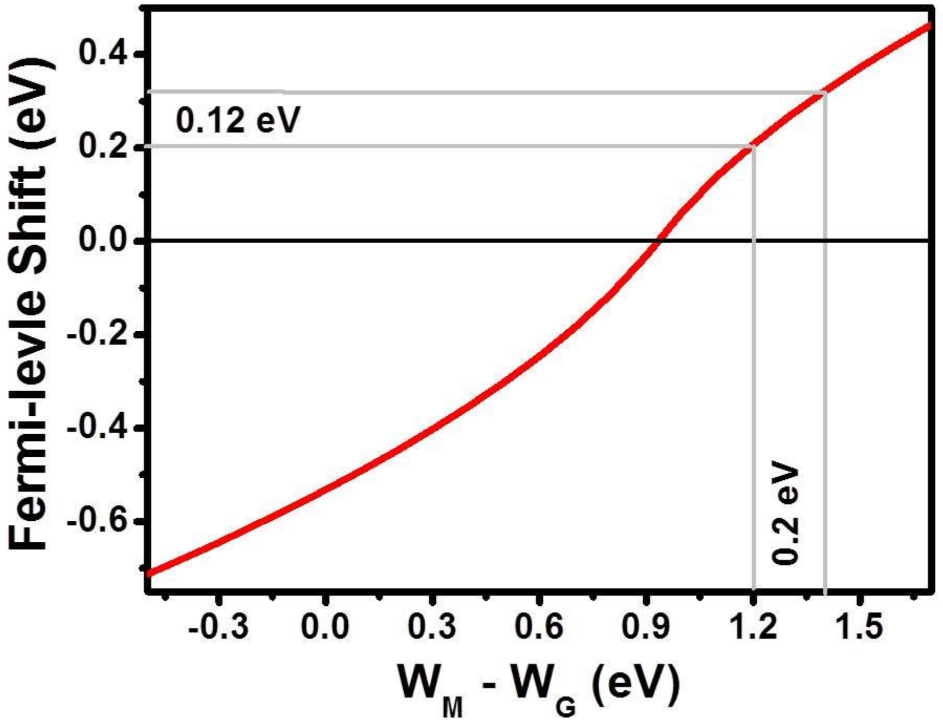

Figure S2: Relationship between work function difference between graphene and and a metal support W_M-W_G and the Fermi-level shift in graphene due to charge transfer. This relationship has the analytical form …. And was derived by xxx et al. by DFT simulations [ref]. Note that charge neutral graphene is not obtained if graphene and the metal have the same work function (W_M-W_G= 0 eV) but if the metal has ~ 0.9 eV larger work function than charge neutral graphene. This behavior is due the formation of an interface dipole due to the 'push-back' effect of the graphene frontier orbitals if adsorbed on a metal. This interface dipole can be described as an effective work function of the substrate that is ~0.9 eV (according to these calculations or ~ 1 eV according to our measurements) lower than the work function of the free surface. We also indicate the expected Fermi-level shift for graphene supported on the 2D-FeO/Pt(111) surface with varying surface potential. For the measured work function variation of ~0.2 eV a shift in the Fermi-level of 0.12 eV is anticipated. This value is used in the manuscript for the separation of the two components of the C-1s peak.
